# Utility of core to peripheral temperature gradient using infrared thermography in the assessment of patients with sepsis and septic shock in the emergency medicine department

**DOI:** 10.1186/s12245-025-00890-8

**Published:** 2025-05-07

**Authors:** Vrinda Lath, Prithvishree Ravindra, Freston Marc Sirur, Rachana Bhat, Avinash Bhat, Karthik Naik, Ramya R, Jayaraj Mymbilly Balakrishnan

**Affiliations:** 1 Department of Emergency Medicine, Kasturba Medical College, Manipal, Manipal Academy of Higher Education, Manipal, Udupi, 576104 Karnataka India; 2 Department of Emergency Medical Technology, Manipal College of Health Professions, Manipal, Manipal Academy of Higher Education, Manipal, Udupi, India

**Keywords:** Sepsis, Infrared thermography, Peripheral perfusion, Septic shock, Microcirculation, Resuscitation

## Abstract

**Objective:**

Sepsis is a disease affecting microcirculation, reflected in temperature changes between the core and the skin. This study explores correlation of this gradient using infrared thermography (IRT) with mortality and markers of hypoperfusion in patients admitted with sepsis and septic shock and its changes with resuscitation.

**Design:**

We conducted a prospective, single center observational study on patients admitted in the Department of Emergency Medicine of a tertiary care center in Karnataka, India. These patients were enrolled based on the inclusion criteria and infrared thermography was performed and cases were followed up after 28 days. Adults presenting to the emergency medicine department with clinically suspected sepsis or septic shock were enrolled and infrared thermography was performed. A final sample size of 187 cases was analyzed after retrospectively excluding patients with any exclusion criteria.

**Interventions:**

Patients underwent thermal imaging of all four limbs on arrival and after 3  hours of resuscitation. Core temperature was measured using a tympanic thermometer. Infrared thermography was performed, and limb temperature was extracted from the images. Other parameters including mean arterial pressure and lactate were recorded and SOFA score was calculated.

**Outcome measure(s):**

The temperature gradients were correlated with 7 and 28-day mortality along with markers of hypoperfusion including mean arterial pressure and serum lactate levels.

**Results:**

A total of 187 patients were included, with a mean SOFA score of 5. Forty four patients (23.5%) died within 7-days. 28-day mortality was 31%. Temperature gradients of core to knee > 8.85°F (*p* = 0.003) and core to great toe > 12.25°F (*p* = 0.020) on arrival were found to be correlated with 7-day mortality. Core to knee temperature gradient was found to correlate with 48-hour mortality(*p* < 0.013). Core to index finger gradient on arrival correlated with vasopressor requirement within 48h (*p* = 0.020). Core to index finger temperature gradient had a negative correlation with mean arterial pressure (spearman coefficient − 0.286, p = < 0.001), and a positive correlation with lactate (0.281, p = < 0.001), SOFA score (0.242, *p* = 0.001), qSOFA score (0.167, *p* = 0.023).

**Conclusions:**

Core-to-knee and core-to-toe temperature gradients using IRT significantly correlate with 7-day mortality. IRT can be a useful adjunct to predict clinical courses in patients with sepsis and septic shock.

**Supplementary Information:**

The online version contains supplementary material available at 10.1186/s12245-025-00890-8.

## Introduction

Sepsis is an abnormal and exaggerated response to infection and carries both a high burden of morbidity and mortality when it progresses to septic shock [1]. Sepsis accounts for approximately 20% of all cause deaths globally [2]. In India, an estimated 11.3 million people suffered from sepsis with 2.9 million deaths in 2017 [3]. The 28-day mortality due to sepsis at the study center has been estimated at 40% [4]. It is a disease that affects people of all age groups and socioeconomic strata, but the highest burden remains in the lower economic strata due to multiple factors including living conditions and early access to advanced healthcare facilities [2]. It is a medical emergency where timely intervention in the form of appropriate antibiotics and resuscitation can result in better survival rates. Many scoring systems have been proposed to diagnose, monitor, and predict outcomes in sepsis patients, but their sensitivity and specificity vary [1, 5]. The SOFA score is a useful tool for screening and prognosticating sepsis [6, 7]. Other scoring systems include LODS, MEWS, NEWS, and SAPS II [1, 5, 8].

As our understanding of pathogenesis deepens, our attention is directed towards microcirculation, which appears to be key to the pathophysiology of sepsis [9, 10]. It is postulated that the severity of sepsis and early detection of deterioration can be made possible by monitoring microcirculation [11]. Many studies have attempted to assess microcirculation via systems such as, the mottling score [12–14], capillary refill time (CRT) [15, 16], near infrared spectroscopy (NIRS) [17], and sublingual microscopy [18] to quantify the severity of sepsis and predict mortality. While the mottling score and CRT are simple to administer and do not require large equipment or training, they have limited applicability in dark-skinned populations [12, 13, 15, 16]. Other techniques are either imprecise or require heavy equipment and are time consuming [17]. A promising marker is the surface thermal distribution pattern in the body. Many studies have shown that peripheral temperature is correlated with shock and mortality but differs in site selection, correlations, and outcomes [14, 19–22].

Our study aimed to correlate the differences between core and peripheral body temperatures via infrared thermography with the mortality and severity of sepsis. We also attempted to study the changes in temperature gradients with resuscitation in this prospective study in patients who presented with sepsis.

## Materials and methods

### Study design

Our study was a single center, prospective, observational study conducted at the Department of Emergency Medicine, at a tertiary healthcare center in India. The study was conducted from October 2021 to October 2022. Patients were screened in the Department of Emergency Medicine, and those satisfying the inclusion criteria were enrolled. As per the Emergency Severity Index triaging system [23] followed in the department, the patients triaged as priorities 1, 2 and 3 were considered for the study. The turnaround time for emergency physician contact is 3 min for these triage categories, and 15 min for the completion of assessment. Data collection was performed by the investigators and monitored. Before enrolment, the Institutional Ethics Committee approval (project number 156–2021) was obtained and the study was registered with the Clinical Trials Registry, India (CTRI/2021/08/035892). A valid written informed consent was obtained. The study did not receive any external funding. Equipment and infrastructure were obtained from the Department of Emergency Medicine.

### Patients

Eligible patients > 18 years of age with clinical, laboratory or image-based evidence of infection [24] who were admitted to the Department of Emergency Medicine with suspected sepsis or septic shock, were screened via the qSOFA score [5] and SIRS criteria [1] and enrolled for infrared thermography upon arrival. Once investigation reports were available, the SOFA score was calculated, and only patients with scores ≥ 2 [5] were considered for further analysis. Septic shock was diagnosed as per the Sepsis 3 guidelines, as a patient with sepsis having a vasopressor requirement despite fluid resuscitation and a lactate concentration greater than 2mmol/L after resuscitation [5]. Patients were excluded if they did not consent to the study or were suffering from any known peripheral vascular disease. Patients with a final diagnosis other than sepsis were retrospectively excluded. The identification of the source of sepsis was based on microbiological evidence, imaging or other positive markers for infection.

By applying the formula for finite population correction, the minimum sample size needed for the study was calculated. We considered the proportion of mortality as 16.3% [20], the expected number of cases of sepsis in the hospital being approximately 1400 in a year, a 95% CI (Zα = 1.96), and precision of 5%. Based on these factors, the required sample size was calculated to be 183. A random sampling method was employed for our study.

### Endpoints

The primary endpoint was the correlation of the core-to-peripheral temperature gradients with 7-day mortality. The secondary endpoints included correlations with 28-day mortality, mean arterial pressure, serum lactate levels, morbidity and changes in gradient during ongoing resuscitation.

### Methodology

The patient was resuscitated as per institutional protocols which are in accordance with the Sepsis 3 guidelines [5]. After enrolment, basic details including age, gender, comorbidities, history, and prehospital care were recorded. Investigations were carried out as per the protocol. Vital signs and urine output were continuously monitored. Echocardiographic reports were collected if available.

Tympanic thermometry was performed with a Braun Thermoscan P6000 thermometer as a surrogate for the core temperature, as it is non-invasive and can easily be used in emergency settings. Infrared thermography was performed using FLIR E8, Teledyne FLIR, Oregon, USA. The infrared camera produces both qualitative and quantitative maps of temperature (thermograms), which were analysed via a rainbow palette with white/red as hot and blue/black as cold. Once operational, the camera automatically calibrates to the ambient temperature. Following this, images were captured at 5 min. at the bedside at 1 m distance, perpendicular to the site of capture after 10 min of arrival in the ER to allow acclimatization and minimize errors in temperature recordings. Images of both the upper limbs (covering the forearm and tips of the index fingers) and lower limbs (covering the knees and tips of the toes) were recorded. Lactate levels were measured during arterial blood gas analysis in accordance with sepsis management protocols. Mean arterial pressure was calculated from blood pressure recorded non-invasively with an oscillometric device and confirmed with an aneroid sphygmomanometer. These investigations, including temperature recordings, were repeated at 3 h when possible, and if we had access to the patients as the patient flow patterns differed during the COVID-19 outbreak season. The initial SOFA score was calculated as blood test analyses were available.

Using the FLIR Tools software, regions of interest (ROIs) were demarcated, via the spot measuring tool, which focused on the distal phalanx of both index fingers, the extremities of both great toes, the inferomedial aspect of both knees and both cubital fossae. These temperature values were tabulated along with the core temperature measurements on Microsoft Excel v2211.

Patients were followed up until 28-days, as per the availability of follow-up data, through hospital records during subsequent visits or telephonically. Other markers of morbidity including the need for vasopressors, mechanical ventilation, hemodialysis, and surgery, were recorded along with the duration of hospital stay, and ICU stay. Culture/sensitivity data and other relevant serology reports were collected. Statistical analysis was performed via SPSS (Statistical Package for Social Sciences) version 21.0, R version 4.4.1, and MedCalc version 19.0.3.

### Materials

#### Infrared camera

The *FLIR E8* camera is a point-and-shoot infrared camera that provides a thermal image with temperature information in each pixel. It has a resolution of 320 × 240 pixels. Its thermal sensitivity/NETD is < 0.06 °C (0.11 °F) / <60 mK, and its field of view (FOV) is 45° × 34°. The minimum focus distance is 0.5 m. It can be used to measure temperatures in the range of -20 to + 250 °C with an accuracy of ± 2 °C (± 3.6 °F). It has a spatial resolution (IFOV) 2.6 mrad, an F-number of 1.5 and an Image frequency of 9 Hz. The image presentation Display 3.0 in. 320 × 240 color liquid crystal displays the image adjustment with manual and automatic adjustment.

#### Tympanic thermometer

The *Braun Thermoscan P6000* is a clinical grade thermometer used for the measurement of human body temperature. It uses infrared to measure the temperature at the tympanic membrane. It uses ExacTemp™ technology to detect probe stability to improve the reliability of measurements and PerfecTemp to overcome errors due to anatomical variations in the ear canal. Thermometry is not affected by ambient temperature, or the presence of moderate cerumen, otitis media or a tympanostomy tube. The device was calibrated at the time of initiation of the study and was serviced annually.

### Statistical analysis

Data was presented as the means and standard deviations or medians with interquartile ranges for continuous variables and as percentages for categorical variables. Since the data were not normally distributed, the Mann Whitney U test was used to compare the medians of two groups. The chi-square test was performed to determine associations between categorical variables, and the odds ratio (OR) was calculated to measure risk. ROC curves were plotted, and the area under the curve (AUC) was calculated along with significance. The Youden index was used to estimate the cut-off values of each temperature gradient and validate their ability to predict mortality at 7 and 28-days. A grey-zone analysis was conducted to determine an optimal cutoff range for 7-day, 28-day and 48-hour mortality based on core-to knee-and core-to-toe temperature gradients at zero hours.

A P value of less than 0.05 was considered significant. The data was entered into MS Excel and the analysis was performed using SPSS version 21.0 and Medcalc version 19.0.3. The grey-zone analysis was done using R version 4.4.1.

## Results

### Patients

**Fig. 1:**
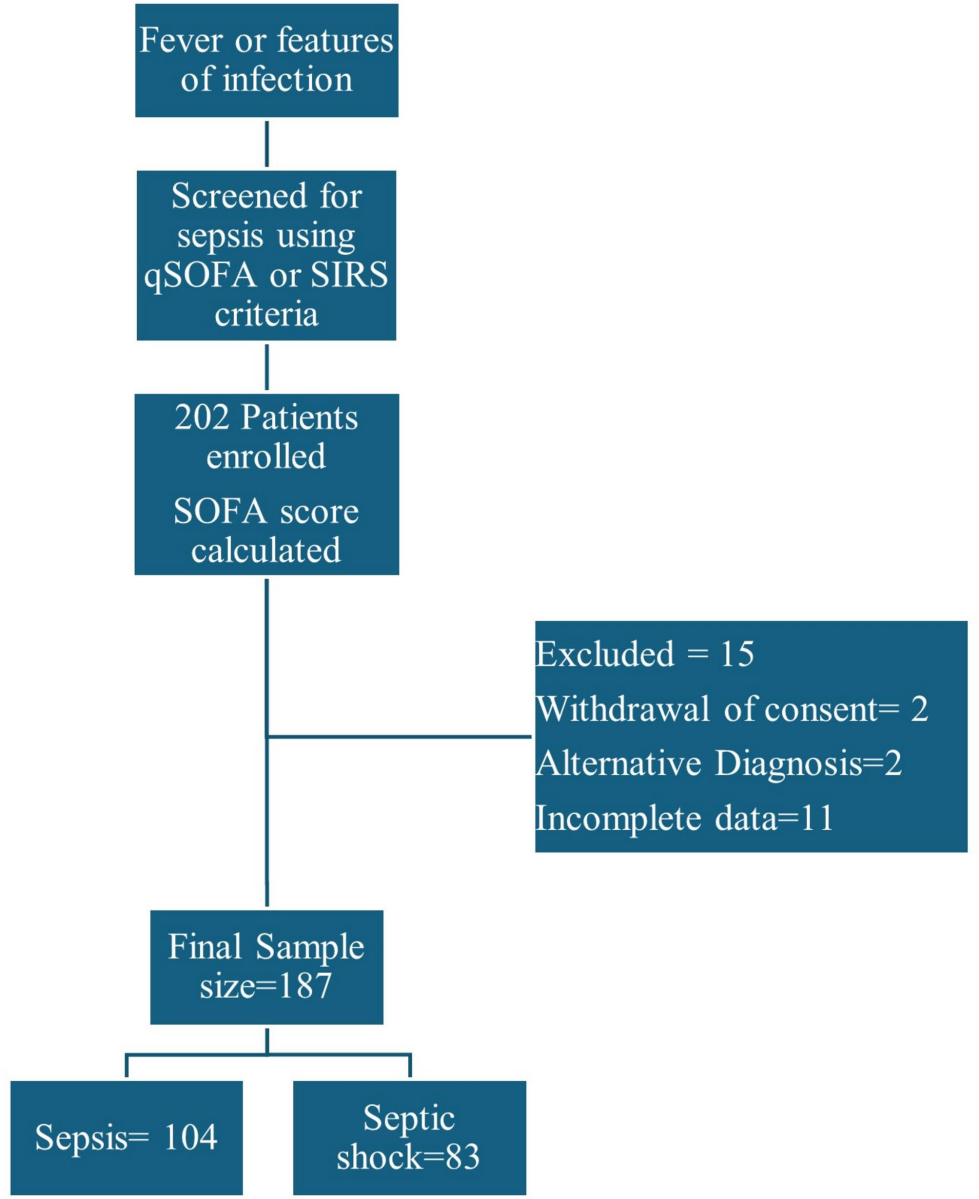
Flow diagram of participants Of 202 patients enrolled after screening, 15 were excluded due to withdrawal of consent, alternative diagnosis and incomplete data. The final number of patients included for analysis was 187 with 104 patients with sepsis and 83 fulfilling the criteria for septic shock

Data collection began in October 2021 and continued until October 2022, and a total of 202 patients were enrolled, 15 of whom were excluded because of the withdrawal of consent and alternative diagnoses (Fig. 1). Among the 187 patients who were followed up, 104 had sepsis (Table S7, Figure S4, S5), and 83 had septic shock (Table S8, Figure S6, S7).

The mean Age was 57.51(15.36) with a minimum of 19 and a maximum of 95 years. A total of 124(66.31%) patients were male. The most common source of sepsis was the respiratory tract 73(39%), followed by tropical fevers (12.2%). (Table 1).

**Table 1:** Baseline characteristics

Characteristics	*N* = 187	Sepsis(*n* = 104)	Septic shock(*n* = 83)
**Age(Mean + SD)**	57.51(15.36)	57.45(15.38)	57.59(15.33)
**Gender(Male)**	66.31%	65.38%	67.46%
**Presenting symptoms(%)**			
Fever	47.1	48.07	45.78
Cough	15.5	18.26	12.04
Shortness of Breath	32.0	33.65	30.12
Fatigue	13.3	13.46	13.25
Altered Mental Status	19.25	18.26	20.48
Limb pain	4.81	3.8	5.95
**Comorbid illness(%)**			
Diabetes mellitus	43.9	48.07	38.55
Hypertension	40.1	47.11	31.32
Chronic kidney disease	7	9.61	3.61
Chronic liver disease	6.4	5.76	7.22
Ischemic heart disease	10.2	9.61	10.84
Lung disease	9.6	13.46	4.81
**Sofa score**	5(3.1)	4 2.25)	6(3.37)
**Therapeutic procedures (%)**			
Vasopressors	44.4	0	100
Intubation	28.3	8.65	55.42
Hemodialysis	9.1	4.87	14.45
**Physiological variables(Mean**,** SD)**			
Mean arterial pressure at 0 h	88.4(18.4)	92.36(14.08)	82.85(19.96)
Lowest mean arterial pressure at 0 h	40	47	37
Highest lactate level	138	110	138
Mean lactate level	31.36(29.15)	22.80(20.95)	41.22(33.72)
Highest WBC count	42,000	42,000	37,800
Lowest platelet level	11,000	12,000	11,000
Highest Bilirubin level	28	28	> 32
Highest Creatinine level	13.8	13.8	10.5
**Primary site of infection(%)**			
Pulmonary	39.03	41.34	33.73
Tropical fever	12.2	13.46	10.84
Skin/soft tissue	11.22	9.61	13.25
Urine	11.22	12.5	9.63
Abdomen	10.69	11.53	9.63
CNS	2.13	2.88	1.20
Catheter	1.06	0.96	1.20
Unknown	12.2	6.73	19.2
**Morbidity Mean (SD)**			
Duration of hospital stay	9.3(8.7)	9.8(7.57)	8.5(9.7)
Duration of ICU stay	5.4(5.3)	4.50(3.94)	6.3(6.3)

While all patients fulfilled the SIRS criteria, the mean q-sofa score was 1.5. All patients had SOFA scores ≥ 2, with a mean SOFA score of 5 and a maximum of 18. Twenty-three patients who expired within 28-days had q-SOFA scores of 1 at presentation.

A total of 44(23.5%) patients expired within 7-days. 22 patients died within 48 h of admission. An additional 14 patients died within 28-days, resulting in an overall 28-day mortality of 31%. A total of 83(44.4%) patients required vasopressors during the first 48 h after admission. Among the patients who died within 7-days, 43 required vasopressors within the first 48 h of admission. Among the patients who died, the mean duration of survival was 9.9 days with a standard deviation of 11.3.

### Thermography

The average ambient temperature was 77.88 °F(72.9–81.5 °F). The mean tympanic temperature and peripheral temperature, as well as the gradients are shown in Table S1 and Table S2. Limb temperatures were calculated by taking an average of the left and right sides. Limbs with local inflammation (cellulitis/necrotizing fasciitis) were excluded from the analysis. A total of 186 patients underwent temperature analysis of the upper limbs whereas 176 patients underwent temperature analysis of the lower limbs.

#### Correlation with mortality

##### 7-day mortality

**Fig. 2:**
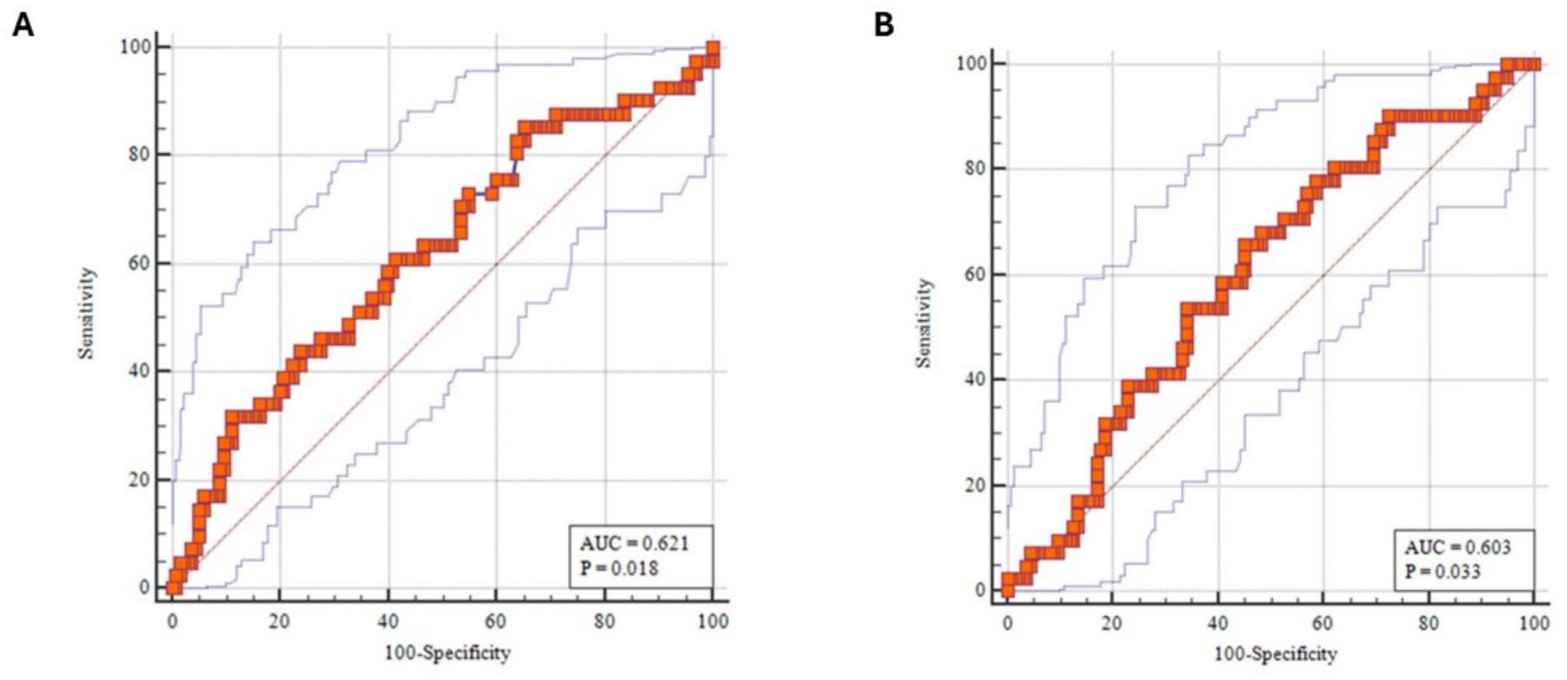
**(a)**: ROC curve correlating core to knee temperature gradient at zero hours with 7-day mortality showing area under curve of 0.621 with a p value of 0.018 **(b)**: ROC curve correlating core to tip of great toe temperature gradient at zero hours with 7-day mortality showing area under curve of 0.603 with a p value of 0.033

**Fig. 3:**
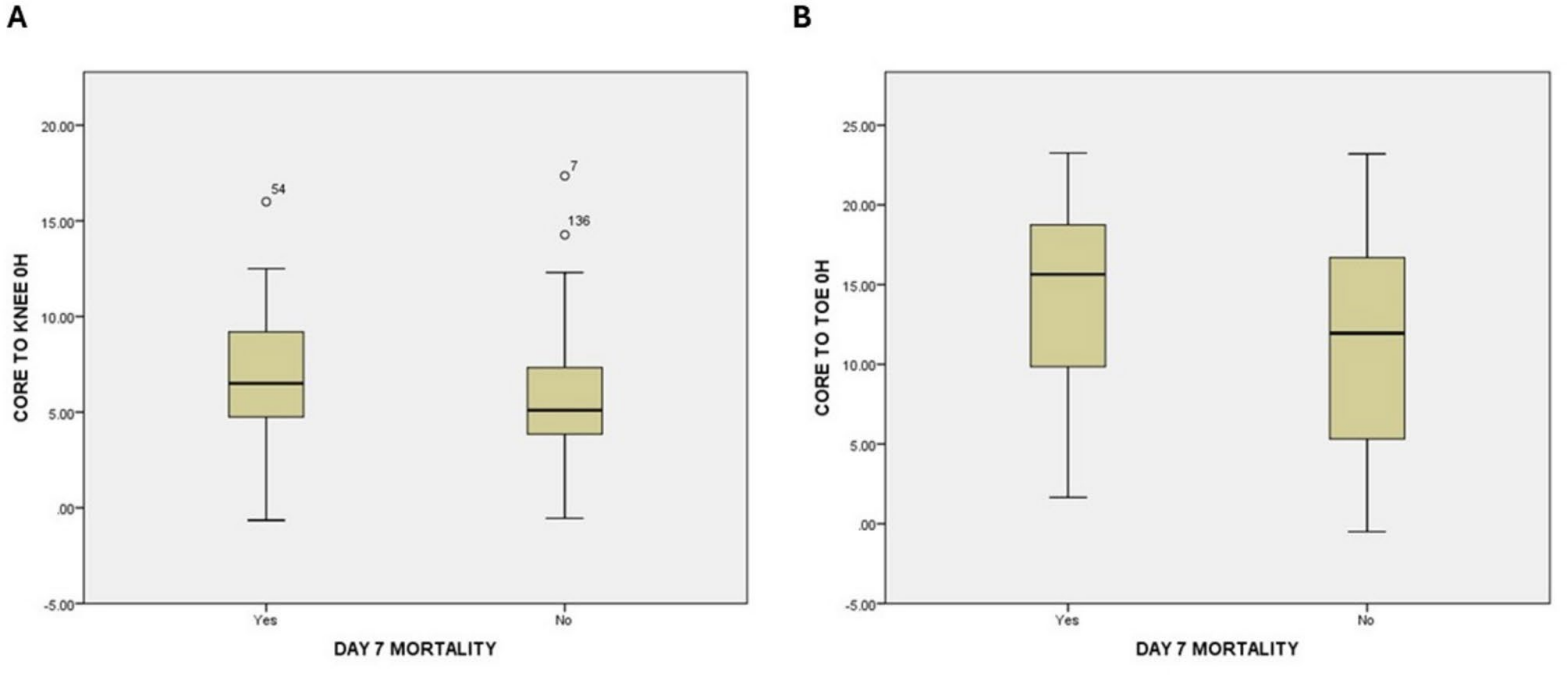
**(a)**: Core to knee temperature gradient at zero hours according to 7-day mortality **(b)**: Core to tip of great toe temperature gradient at zero hours according to 7-day mortality

The Mann Whitney U test revealed that the core-to-knee(*p* = 0.019) and core-to great-toe(*p* = 0.045) temperature gradients at arrival correlated with 7-day mortality. (Table 2). The ROC curves were the highest for the core-to-knee (0.62, *p* = 0.018) (Figs. 2 and 3 (a)) followed by the core-to-great toe (AUC 0.60, *p* = 0.033) (Figs. 2 and 3 (b). A core-to-knee gradient > 8.85 °F (Table 3) had a specificity of 88.2% with a sensitivity of 31.7% and an accuracy of 75% (Table 4).

**Table 2:** Test for correlation of core to peripheral temperature gradient at zero hours with 7-day and 48 h mortality

Temperature Gradient(°F)	DAY 7 MORTALITY						Mann Whitney U test *p* value
	Yes			No			
	Median	Q1	Q3	Median	Q1	Q3	
CORE TO CUBITAL 0 H	3.35	2.25	5.75	3.36	1.60	5.10	0.293
CORE TO INDEX 0 H	8.85	4.65	15.30	7.50	3.95	13.45	0.094
CORE TO KNEE 0 H	6.50	4.75	9.20	5.10	3.85	7.35	**0.019**
CORE TO TOE 0 H	15.65	9.85	18.75	11.95	5.30	16.70	**0.045**
Temperature Gradient(°F)	48 h MORTALITY						Mann Whitney U test p value
	Yes			No			
	Median	Q1	Q3	Median	Q1	Q3	
CORE TO CUBITAL 0 H	4.33	2.05	5.45	3.35	1.70	5.20	0.421
CORE TO INDEX 0 H	11.58	4.05	16.55	7.43	4.03	13.63	0.123
CORE TO KNEE 0 H	7.88	4.95	9.50	5.12	3.95	7.35	**0.013**
CORE TO TOE 0 H	16.53	9.85	18.80	12.43	5.50	16.70	0.093

**Table 3:** Cut-off value of core to limb temperature gradient at zero hours correlating with 7-day mortality

Temperature gradient at 0 h(°F)		DAY 7 MORTALITY						Chisquare test *P* value
		Yes		No		Total		
		N	%	N	%	N	%	
Core to Cubital	> 1.35	40	26.7%	110	73.3%	150	100.0%	0.019 OR = 4.0 (1.16–13.77)
	</=1.35	3	8.3%	33	91.7%	36	100.0%	
Core to Index	> 13.45	18	34.0%	35	66.0%	53	100.0%	0.027 OR = 2.2 (1.09–4.55)
	</=13.45	25	18.8%	108	81.2%	133	100.0%	
Core to Knee	> 8.85	13	44.8%	16	55.2%	29	100.0%	0.003 OR = 3.5 (1.5-8.0)
	</=8.85	28	19.0%	119	81.0%	147	100.0%	
Core to Toe	> 12.45	27	30.7%	61	69.3%	88	100.0%	0.020 OR = 2.3 (1.13–4.9)
	</=12.45	14	15.9%	74	84.1%	88	100.0%	

**Table 4:** Performance of core to peripheral temperature gradients in predicting 7-day mortality

Variables	AUC	Cut off(°F)	Sensitivity	Specificity	PPV	NPV	Accuracy
Core to Cubital	0.553	> 1.35	93.0	23.1	26.7	91.7	39.3
Core to Index	0.584	> 13.45	41.9	75.5	34.0	81.2	67.7
Core to Knee	0.621	> 8.85	31.7	88.2	44.8	81.0	75.0
Core to Toe	0.603	> 12.45	65.9	54.8	30.7	84.1	57.4

##### 48-hour mortality

The core-to-knee temperature gradient - significantly correlated with 48-hour mortality, with a median value of 7.88 °F (*p* < 0.013) (Table 2).

##### 28-day mortality

None of the gradients were significantly correlated with 28-day mortality (Table S3).

#### Correlations with other markers of hypoperfusion and morbidity

A total of 45 patients were started on vasopressors in the Emergency Medicine Department, of whom 4 patients received vasopressors from the referring centers. The core-to-index finger gradient at arrival correlated with the vasopressor requirement within 48 h (*p* = 0.020) (Table 5, Figure S1).The core-to-index finger temperature gradient was having a negative correlation with the mean arterial pressure (Spearman coefficient − 0.286, p = < 0.001), and positively correlated with the lactate level (0.281, p = < 0.001)(Figure S2), SOFA score (0.242, *p* = 0.001), qSOFA score (0.167, *p* = 0.023). The Mann- Whitney U test revealed a correlation between the 0-hour core-to-great toe gradient and the lactate level, and the duration of ICU stay (Figure S3) (Table S4). While all the parameters were significantly correlated with the vasopressor requirement within 48 h, there was no statistically significant correlation between lactate clearance and the change in the temperature gradient between 0 and 3 h (Table S6).

**Table 5:** Correlation of core to peripheral temperature gradient at zero hours with vasopressor requirement within 48 h

Temperature Gradient(°F)	Vasopressors						Mann Whitney U test *p* value
	Yes			No			
	Median	Q1	Q3	Median	Q1	Q3	
CORE TO CUBITAL 0 H	3.45	2.05	5.55	3.33	1.45	5.00	0.212
CORE TO INDEX 0 H	8.85	4.60	15.85	7.28	3.93	12.10	**0.020**
CORE TO KNEE 0 H	5.25	4.20	8.25	5.20	3.90	7.35	0.440
CORE TO TOE 0 H	13.65	6.70	19.20	11.80	5.30	16.60	0.066

## Discussion

Despite rapid advances in the management of sepsis, mortality in patients with septic shock remains high. The search for markers of hypoperfusion and mortality in sepsis patients is still a subject of ongoing research. An ideal marker should be inexpensive, easy to administer, safe, reliable, and easily available [25]. One potential point-of-care tool that is rapid, noninvasive, and requires minimal training is infrared thermography [26].

### Site selection

Site selection was based on data available in previous studies suggesting that the earliest signs of microcirculatory dysfunction may be detected in the extremities [9, 12–14, 20, 21]. The tips of the index finger, great toe and dorsum of the knee are sites for measuring the CRT, and skin mottling begins at the knee [13, 15]. These sites, supplied by small vessels, are accessible and the most studied for microvascular derangements [13–16]. For these reasons, we selected 4 sites for measuring the peripheral temperature- the cubital fossa, the tip of index finger, the inferomedial aspect of knee and the tip of the great toe [20]. Tympanic temperature has been reported to be comparable with esophageal temperatures [27], but is limited by the risk of measurement error, similar to nasopharyngeal temperature. Other methods, such as rectal and vesical temperature measurements, while being good measures, are subject to high latency, and subject the patient to discomfort. The most reliable measures are at the pulmonary artery and esophagus, both of which are limited by the risk of complications [27]. Tympanic temperature was chosen for this study as a less invasive method to measure the core temperature, despite some variability in real time Emergency care settings [20, 27].

### Clinical and demographic details

Most of our patients were between 51 and 70 years of age, similar to Rhee et al., who reported that the mean age was 70.5 years [28]. This could be attributed to comorbid diseases increasing the risk of infection. According to a report by the WHO, the incidence of sepsis is biphasic, with the second peak occurring in older adults [2].

Notably, only 88 patients (47.1%) presented with fever. Many patients were febrile at presentation but had not noticed or recorded a fever previously. Immunocompromised and diabetic patients may not develop fever. Our findings are consistent with the findings of Kushimoto et al., who reported that only 39% of patients presented with fever [29].

### Scoring systems

While the q-SOFA score and SIRS criteria were used to screen patients on arrival, the limitations of these scoring systems were considered, both in terms of specificity (q-SOFA) and sensitivity (SIRS) [8, 30]. While our center is yet to be equipped with machine learning, we used a combination of clinical features of infection, q-SOFA score and SIRS to screen for patients with sepsis and confirm with the SOFA score once the parameters were available. Notably, although all patients had SOFA scores > 2, 50.8% of the patients had a q-SOFA score of 1. Compared with other tools, the updated surviving sepsis guidelines recommend against q-SOFA score as a single screening test for sepsis [1]. Our results also suggest that the q-SOFA cannot be used as a standalone screening tool in the Emergency Department for sepsis. There is a need to develop a robust tool that is both sensitive and specific in the emergency setting to screen for sepsis, and IRT may still be a useful adjunct. CRT and Mottling were not included in this study because of their limitations in our study population [12, 15, 16].

2*Mortality correlation*: Core-to-knee and core-to-great toe temperature gradients at ED presentation were found to be significantly correlated with 7-day mortality[ 2, 28, 29 ]The core-to-knee temperature was also correlated with 48-hour mortality. Among the four gradients, the core-knee temperature had the highest specificity and accuracy for predicting 7-day mortality. Multiple studies have used the knee as a window into microcirculation [12–14, 20]. It may be possible that knee temperature alone may indicate shock and predict mortality. The sympathetically innervated vasculature may contribute to this [9, 13, 31, 32]. With infrared thermography, the site appears cooler, appearing yellow or blue on the color palette, which may be used as a rapid assessment tool when precise metrics are unavailable. Amson et al. used a similar methodology in patients with septic shock and concluded that the core- to-index finger gradient had the highest correlation with 8-day mortality [20]. Other studies reported significant correlations with great toe temperatures [21, 22, 33]. Our study revealed a lower sensitivity and higher specificity of IRT in predicting 7-day mortality.

Compared with 28-day mortality all 4 sites showed poor discrimination with AUCs between 0.5 and 0.6 and P values > 0.05. This result is consistent with other studies correlating with 28-day mortality [14, 34]. We used a shorter duration of survival as a primary outcome as it reduces the contribution of other factors such as complications during the ICU stay, comorbidities, vascular events, and hospital-acquired infections to mortality [20].This cross sectional study uses a point-of-care tool to evaluate a dynamic parameter that is more indicative of an acute physiology, and our results prove the primary purpose of the tool. The temperature gradient is therefore assumed to be a poor predictor of 28-day mortality. It is known that sepsis and septic shock increase the risk of further infection [2]. Impaired peripheral circulation may contribute to susceptibility to disease. There are multiple confounding factors that compound the risk of mortality at 28-days and later.

According to the gray zone analysis, none of the thresholds reached a 95% rule in probability, with a high percentage of values falling within the gray zone. The identified ranges highlight uncertainty in classification, suggesting that the gradients cannot be used as a standalone threshold to predict mortality. However, they may still be used as adjuncts to other tools (Figure S8, S9, S10, S11, Table S9, S10).

While the correlation appears to be poor compared with the Mottling score [12], and CRT [15] in predicting mortality, the results of our study are comparable with those of the ARISTOS study using NIRS [17]. However, these methods also have many limitations. CRT is limited by subjectivity, technique, ambient conditions, patient conditions and skin conditions. The mottling score cannot be used in dark skinned individuals [12–14]. NIRS is limited by the cost and availability of the device [17]. Body surface thermal inhomogeneity parameters may be a more reliable marker of critical illness, where a low temperature area rate of more than 10% of the maximum temperature has been reported to be a comprehensive marker of hypoperfusion [35]. Larger analyses of thermal inhomogeneity patterns may be needed to identify the most reliable technique of assessing hypoperfusion.

### Correlation with other markers of hypoperfusion and morbidity

The initial core-to-index finger temperature gradient correlated significantly with vasopressor requirements. Although all the parameters at 3 h significantly correlated with the vasopressor requirement, the clinical significance needs to be further studied to determine the effect of vasopressors on circulation. The use of vasopressors as a contributing factor could not be excluded, as this was pragmatically studied to assess its utility as a real-time tool in patients, particularly those with septic shock who invariably receive vasopressors.

The core to index finger at 0 h and the core to cubital and index finger at 3 h were found to have a weak negative correlation with the mean arterial pressure. Amson et al. reported that the correlation was very weak [20]. This is possibly because microcirculatory changes do not always affect macro-circulation, for which the mean arterial pressure is a measure [10]. While microcirculatory changes often preceded macro-circulatory changes, the correlation may be better demonstrated in patients with septic shock. BP recordings were performed manually via an aneroid sphygmomanometer and non-invasively using an oscillometric device. This is not the gold standard of measurement and is subject to operator errors. Perhaps arterial blood pressure monitoring may provide a clearer correlation with these parameters.

While the core-to-index and core-to-great toe temperature gradients were correlated with lactate levels at 0 h, the core-to-cubital and core-to-great toe correlate with lactate levels at 3 h. However, a change in the gradient was not found to be correlated with lactate clearance at 0 and 3 h [33]. Possible reasons for this could be the inclusion of patients without shock. Other reasons could be the timing of thermography and lactate measurement. IRT may measure different aspects of microcirculation and therefore may not be correlated. A more robust study design and frequent measurements may be needed to establish a correlation between the two parameters.

A Mann-Whitney U test performed for temperature gradients at 0 h and the duration of ICU stay revealed a significant correlation with the core-to-great toe temperature gradient. A core-to-toe temperature gradient may aid in the identification of patients requiring critical care as well as prognostication.

The SOFA score was found to be weakly correlated with the core-to-index gradient at 0 h and the core-to-cubital gradient at 3 h. Amson et al. reported a strong correlation with the SOFA score [20]. One of the possible reasons was the inclusion of patients without shock.

Limitations.

Sampling: As only 91 patients could undergo thermography at 3 h, significant inferences could not be drawn. Reproducibility: The generalizability of the results cannot be commented upon as the study was performed in a single ED setting. Core temperature: Tympanic thermometry is not the most accurate measure of core temperature but was used because it is the least invasive of the different modalities. Bias: There is a possibility of selection bias as cases were screened for infection by treating clinicians on the basis of clinical signs and symptoms. While vasopressors may have affected thermography findings, more detailed studies are needed to understand the effects. Patients on vasopressors were not excluded from our study to pragmatically understand the utility of IRT as a real-time tool. Similarly, IV fluids and drugs may influence thermography. Our emergency department is centrally air-conditioned, with an overall variability in temperature of 72.9 °F to 81.5 °F. There is no data available on gradients in a normal population for comparison [36]. The infrared camera is not specifically designed for medical applications.

**Fig. 4:**
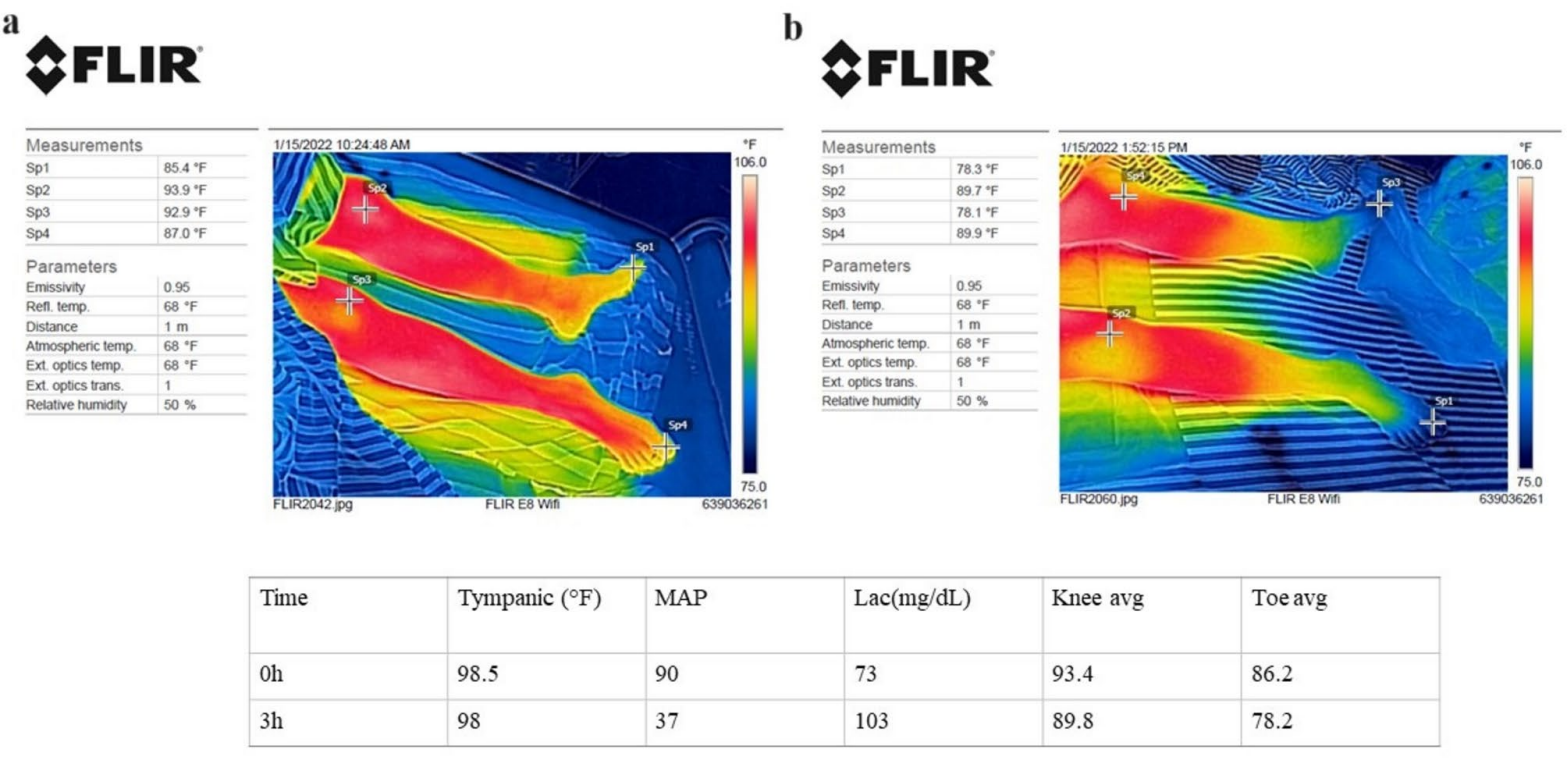
Representative infrared images of a 23-year-old female admitted with septic shock at zero (**a**) and 3 h (**b**) with ROIs marked. The patient passed away within 48 h of admission MAP- Mean arterial pressure; Knee avg, Toe avg- average temperatures of both knees and great toes, represented in °F

Role of infrared thermography: While many studies have attempted to use temperature as a method of monitoring microcirculatory changes in sepsis patients, some have used IRT for the detection of shock, all of which have shown that IRT is a potential tool for monitoring critically ill patients. We chose infrared thermography to monitor skin temperature as it is a non-invasive, non-contact method of measurement and provides visual/ subjective evidence of the temperature expressed as color, thus making it a rapid, effective tool to gauge the gradient in real-time. An added advantage of non-contact methods such as IRT was obvious during the COVID-19 pandemic where IR-based thermometers were used in public spaces for screening for fever. It is also advantageous in the emergency setting as a handheld or a mounted device that provides a real-time, colour gradient image that can be used for rapid screening in a setting where time may be a constraint. Paired with machine learning, it can be a powerful tool for detecting shock and prognostication. (Fig. 4)

Another potential application of IRT may be in pre-hospital settings, where it may be used for rapid screening for shock and for screening a large population in a disaster setting.

## Conclusion

The core-to-knee and core-to-toe temperature gradients determined via infrared thermography were correlated with 7-day mortality in patients with sepsis. Infrared thermography can be a good adjunct for rapidly assessing circulation and predicting clinical deterioration in patients with sepsis.

## Electronic supplementary material

Below is the link to the electronic supplementary material.

Supplementary Material 1

## Data Availability

The datasets generated and/or analysed during the current study are not publicly available to maintain patient confidentiality but are available from the corresponding author on reasonable request.

## Abbreviations

SOFA: Sequential Organ Failure Assessment
MEWS: Modified Early Warning Score
NEWS: National Early Warning Score
SAPS II: Simplified Acute Physiology Score II
LODS: Logistic Organ Dysfunction Score
CRT: Capillary Refill Time
NIRS: Near Infrared Spectroscopy
NO: Nitric Oxide
qSOFA: Quick Sequential Organ Failure Score
SIRS: Systemic Inflammatory Response Syndrome
ROI: Region of Interest
NETD: Noise Equivalent Temperature Difference
IFOV: Instantaneous Field of View
FOV: Field of View
ICU: Intensive Care Unit
ED: Emergency Department
HR: Heart Rate
RR: Respiratory Rate
PaCO2: Partial Pressure of Arterial Carbon Dioxide
PaO2: Partial pressure of oxygen in arterial blood
WBC: White Blood Cell
GCS: Glasgow Coma Scale
MAP: Mean Arterial Pressure
BP: Blood Pressure
pO2: Partial Pressure of Oxygen
FiO2: Fraction of Inspired Oxygen
ROC curve: Receiver Operating Characteristic Curve
IRT: Infrared Thermography
ROI: Region of Interest
AUC: Area Under the ROC curve
