# Supplementary material for: Utility of core to peripheral temperature gradient using infrared thermography in the assessment of patients with sepsis and septic shock in the emergency medicine department
Source: Int J Emerg Med. 2025 May 7;18:93. doi: 10.1186/s12245-025-00890-8 (PMC12057086; doi:10.1186/s12245-025-00890-8)
Supplement: Supplementary file 1 — Supplementary Material 1 [file 12245_2025_890_MOESM1_ESM.docx]

**Table S1:** Mean peripheral temperatures in sepsis and septic shock.

| Temperature(°F) | Mean | SD | Minimum | Maximum |
| --- | --- | --- | --- | --- |
| “zero” hours | | | | |
| TYMPANIC 0H(N=186) | 99.37 | 1.70 | 95.50 | 105.90 |
| CUBITAL 0H(N=186) | 95.66 | 3.11 | 86.35 | 103.50 |
| INDEX 0H(N=186) | 90.54 | 6.16 | 76.60 | 101.50 |
| KNEE 0H(N=176) | 93.45 | 3.35 | 83.80 | 100.45 |
| TOE 0H(N=176) | 87.30 | 6.76 | 75.50 | 100.10 |
| 3 hours | | | | |
| Temperature(°F) | Mean | SD | Minimum | Maximum |
| TYMPANIC 3H (90) | 98.45 | 1.41 | 95.40 | 103.50 |
| CUBITAL 3H(N=90) | 94.99 | 2.84 | 87.50 | 101.50 |
| INDEX 3H(N=90) | 92.64 | 3.26 | 84.70 | 99.15 |
| KNEE 3H(N=89) | 92.64 | 3.26 | 84.70 | 99.15 |
| TOE 3H(N=89) | 86.66 | 7.09 | 72.45 | 99.70 |

**Table S2:** Core to limb temperature gradients at 0 and 3 hours

| Temperature Gradient(°F) | Mean | SD | Minimum | Maximum | Median | Q1 | Q3 |
| --- | --- | --- | --- | --- | --- | --- | --- |
| CORE TO CUBITAL 0H | 3.72 | 2.78 | -2.10 | 15.05 | 3.36 | 1.80 | 5.25 |
| CORE TO INDEX 0H | 8.80 | 5.89 | -2.40 | 24.16 | 7.60 | 4.05 | 13.95 |
| CORE TO KNEE 0H | 5.92 | 3.00 | -.65 | 17.35 | 5.25 | 4.05 | 7.81 |
| CORE TO TOE 0H | 12.07 | 6.54 | -.50 | 23.25 | 12.53 | 5.78 | 17.41 |
| Temperature Gradient(°F) | Mean | SD | Minimum | Maximum | Median | Q1 | Q3 |
| CORE TO CUBITAL 3H | 3.46 | 2.69 | -2.35 | 14.30 | 3.40 | 1.70 | 4.90 |
| CORE TO INDEX 3H | 8.84 | 7.09 | -2.30 | 24.30 | 8.46 | 2.40 | 15.45 |
| CORE TO KNEE 3H | 5.81 | 3.16 | .10 | 16.65 | 5.20 | 3.40 | 8.20 |
| CORE TO TOE 3H | 11.80 | 7.17 | -.90 | 26.85 | 12.60 | 5.35 | 18.30 |

**Table S3:** Correlation of core to peripheral temperature gradient at zero hours with 28-day mortality

| Variables | AUC | Cut off | Sensitivity | Specificity | PPV | NPV | Accuracy |
| --- | --- | --- | --- | --- | --- | --- | --- |
| Core to Elbow | 0.552 | >1.35 | 89.5 | 23.3 | 34.0 | 83.3 | 43.6 |
| Core to Index | 0.522 | >14.06 | 29.8 | 78.3 | 37.8 | 71.6 | 63.4 |
| Core to Knee | 0.589 | >4.8 | 69.1 | 46.3 | 36.9 | 76.7 | 53.4 |
| Core to Toe | 0.550 | >12.1 | 61.8 | 50.4 | 36.2 | 74.4 | 54.0 |

**Table S4(a)**: Correlation of core to peripheral temperature gradient at zero hours with other parameters

| Variables | Core to Cubital | | Core to Index | | Core to Knee | | Core to Toe | |
| --- | --- | --- | --- | --- | --- | --- | --- | --- |
|  | Spearman | P value | Spearman | P value | Spearman | P value | Spearman | P value |
| q-SOFA | 0.117 | 0.112 | 0.167 | **0.023** | 0.069 | 0.361 | 0.045 | 0.550 |
| SOFA | 0.074 | 0.317 | 0.242 | **0.001** | 0.053 | 0.486 | 0.134 | 0.076 |
| Hospital duration | 0.050 | 0.495 | -0.019 | 0.800 | -0.064 | 0.398 | 0.019 | 0.803 |
| ICU duration | 0.090 | 0.227 | 0.014 | 0.857 | -0.052 | 0.496 | -0.001 | 0.986 |
| Lactate | 0.096 | 0.190 | 0.281 | **<0.001** | 0.103 | 0.174 | 0.243 | **0.001** |
| MAP | -0.119 | 0.105 | -0.286 | **<0.001** | -0.010 | 0.891 | -0.043 | 0.575 |

**Table S4(b)**: Correlation of core to peripheral temperature gradient at three hours with other parameters

| Variables | Core to Elbow | | Core to Index | | Core to Knee | | Core to Toe | |
| --- | --- | --- | --- | --- | --- | --- | --- | --- |
|  | Spearman | P value | Spearman | P value | Spearman | P value | Spearman | P value |
| Age | 0.089 | 0.402 | 0.178 | 0.094 | 0.197 | 0.065 | 0.144 | 0.177 |
| qSOFA | 0.107 | 0.313 | 0.171 | 0.106 | -0.036 | 0.737 | 0.121 | 0.261 |
| SOFA | 0.237 | **0.024** | 0.162 | 0.126 | 0.042 | 0.699 | 0.151 | 0.159 |
| Hospital duration | -0.067 | 0.531 | -0.008 | 0.938 | -0.160 | 0.135 | 0.026 | 0.811 |
| ICU duration | -0.055 | 0.612 | 0.016 | 0.884 | -0.109 | 0.311 | 0.100 | 0.355 |
| Lactate | 0.319 | **0.004** | 0.208 | 0.064 | 0.152 | 0.180 | 0.246 | **0.029** |
| MAP | -0.299 | **0.004** | -0.314 | **0.003** | -0.017 | 0.871 | -0.168 | 0.115 |
| Lactate clearance | -0.047 | 0.676 | -0.053 | 0.640 | 0.018 | 0.874 | 0.001 | 0.990 |

**Table S5**: Correlation of core to peripheral temperature gradient with vasopressor requirement within 48 hours

| Temperature Gradient | “Zero hours” | | | | | | Mann Whitney U test  p value |
| --- | --- | --- | --- | --- | --- | --- | --- |
|  | Yes | | | No | | |  |
|  | Median | Q1 | Q3 | Median | Q1 | Q3 |  |
| CORE TO ELBOW 0H | 3.45 | 2.05 | 5.55 | 3.33 | 1.45 | 5.00 | 0.212 |
| CORE TO INDEX 0H | 8.85 | 4.60 | 15.85 | 7.28 | 3.93 | 12.10 | **0.020** |
| CORE TO KNEE 0H | 5.25 | 4.20 | 8.25 | 5.20 | 3.90 | 7.35 | 0.440 |
| CORE TO TOE 0H | 13.65 | 6.70 | 19.20 | 11.80 | 5.30 | 16.60 | 0.066 |
| Temperature Gradient | 3 hours | | | | | | Mann Whitney U test  p value |
|  | Yes | | | No | | |  |
|  | Median | Q1 | Q3 | Median | Q1 | Q3 |  |
| CORE TO ELBOW 3H | 4.30 | 2.75 | 5.70 | 2.55 | 1.10 | 3.70 | **<0.001** |
| CORE TO INDEX 3H | 10.70 | 3.55 | 17.45 | 5.35 | 2.40 | 13.60 | **0.037** |
| CORE TO KNEE 3H | 5.95 | 3.98 | 8.63 | 4.55 | 3.30 | 7.30 | **0.047** |
| CORE TO TOE 3H | 17.40 | 6.40 | 20.03 | 8.30 | 4.81 | 15.20 | **0.013** |

**Figure S1**: Core to tip of index finger temperature gradient at zero hours according to vasopressor requirement within 48 hours

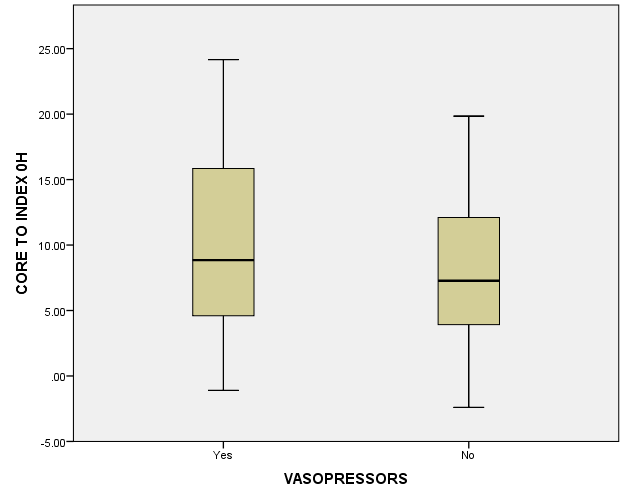


**Figure S2**: Core to tip of index finger temperature gradient at zero hours according to lactate level at zero hours


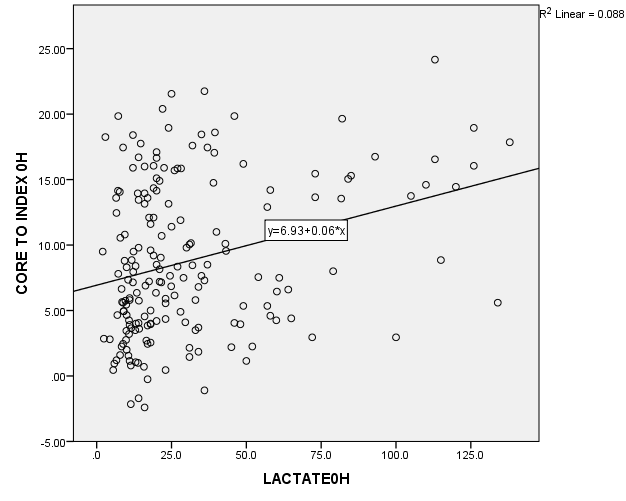


**Figure S3**: Core to tip of great toe temperature gradient at zero hours according to lactate level at zero hours


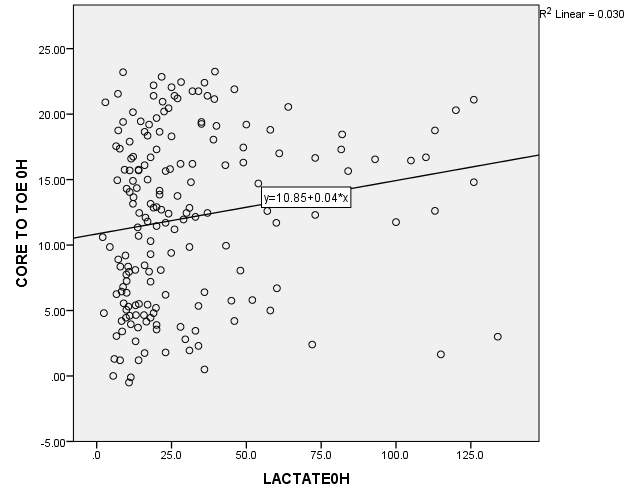


**Table S6**: Correlation of lactate clearance with delta gradient

| Variables | Spearman correlation | P value |
| --- | --- | --- |
| Core to Elbow | 0.129 | 0.259 |
| Core to Index | 0.114 | 0.319 |
| Core to Knee | 0.030 | 0.793 |
| Core to Toe | 0.024 | 0.837 |

**SUBANALYSIS OF SEPSIS AND SEPTIC SHOCK**

**SEPSIS**

**Table S7:** Correlation of gradients with 7-day mortality in patients with sepsis

|  | 7 DAY MORTALITY | | | | | |  |
| --- | --- | --- | --- | --- | --- | --- | --- |
|  | Yes | | | No | | |  |
|  | Median | Percentile 25 | Percentile 75 | Median | Percentile 25 | Percentile 75 | Significance |
| CORE-ELBOW | 4.15 | 4.15 | 4.15 | 3.25 | 1.35 | 4.90 | .673^c^ |
| CORE-INDEX | 13.95 | 13.95 | 13.95 | 7.35 | 3.90 | 12.45 | .385^c^ |
| CORE-KNEE | 4.80 | 4.80 | 4.80 | 5.20 | 3.90 | 7.35 | .788^c^ |
| CORE-TOE | 5.55 | 5.55 | 5.55 | 11.40 | 8.90 | 14.00 | .212^c^ |

**Figure S4:** Scatter plot depicting Core to knee temperature gradient at zero hours with duration of survival


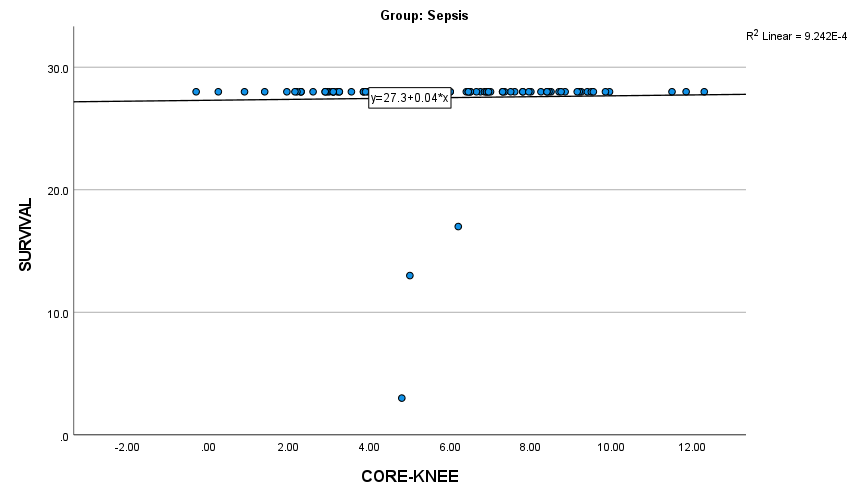


**Figure S5:** Scatter plot depicting core to toe temperature gradient at zero hours with duration of survival


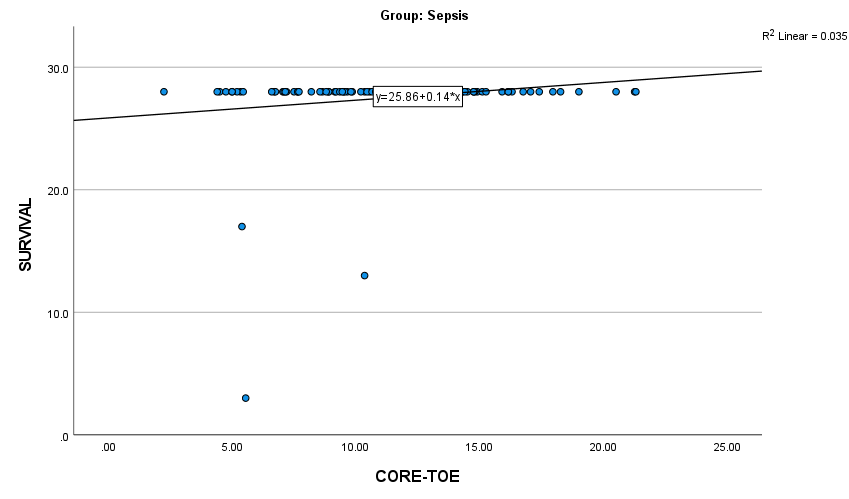


**SEPTIC SHOCK**

**Table S8:** Correlation of gradients with 7-day mortality in patients with septic shock

|  | 7 DAY MORTALITY | | | | | |  |
| --- | --- | --- | --- | --- | --- | --- | --- |
|  | Yes | | | No | | |  |
|  | Median | Percentile 25 | Percentile 75 | Median | Percentile 25 | Percentile 75 | Significance |
| CORE-ELBOW | 3.00 | 2.05 | 5.45 | 3.77 | 1.70 | 6.05 | .802 |
| CORE-INDEX | 8.85 | 4.65 | 15.40 | 8.67 | 4.58 | 15.90 | .639 |
| CORE-KNEE | 6.35 | 4.60 | 9.20 | 4.87 | 3.63 | 7.59 | .061 |
| CORE-TOE | 12.80 | 9.10 | 16.10 | 10.55 | 7.35 | 15.20 | .166 |

**Figure S6:** Scatter plot depicting core to knee temperature gradient at zero hours with duration of survival


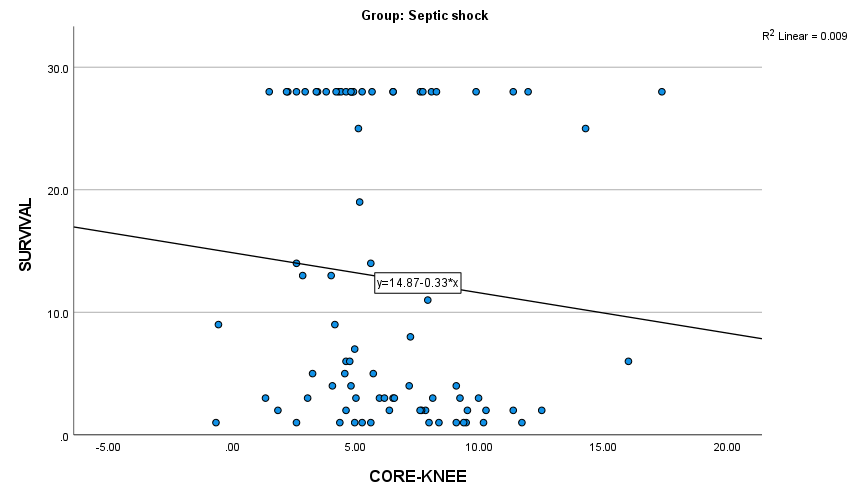


**Figure S7:** Scatter plot depicting core to toe temperature gradient at zero hours with duration of survival


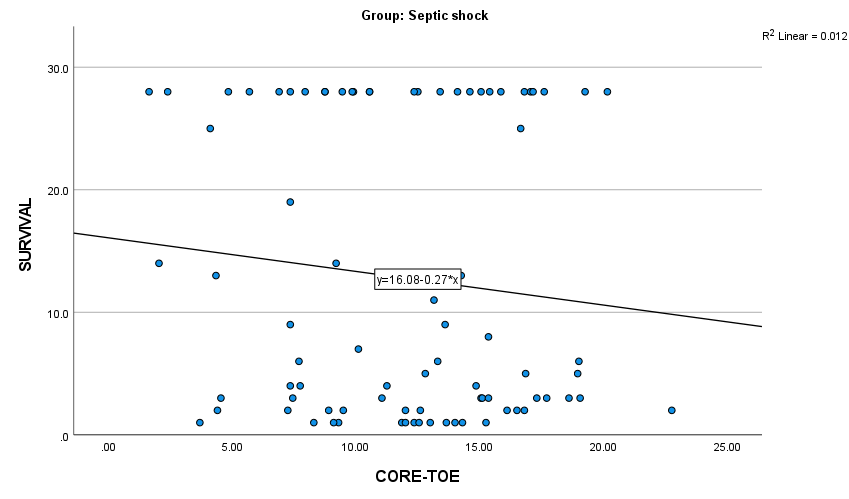


**SURVIVAL ANALYSIS**

**Figure S8:** Overall survival probability using core to knee temperature gradient at zero hours with cutoff of 4.9°F. Patients with **core to knee temperature >4.9** had a shorter survival time. A **Log-rank test p-value** of 0.066 Suggests a **trend towards significance** but does not meet the threshold (P < 0.05) for statistical significance.


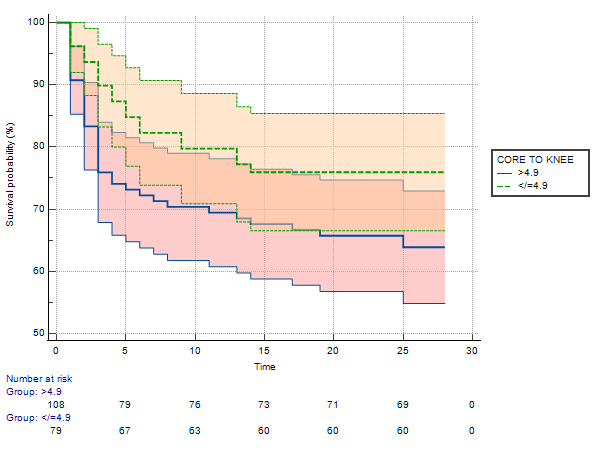


**Table S9:** Mean survival times in all patients taking a zero-hour cutoff of core to knee temperature gradient at 4.9°F.

| Core to Knee | Mean survival time | SE | 95% CI for the mean |
| --- | --- | --- | --- |
| >4.9 | 19.815 | 1.111 | 17.637 to 21.992 |
| </=4.9 | 22.582 | 1.107 | 20.414 to 24.751 |
| Overall | 20.984 | 0.800 | 19.416 to 22.552 |

**Figure S9: S**urvival probability analysis in patients with septic shock using core to knee temperature gradient at zero hours with cutoff of 4.9°F. Patients with **core to knee temperature >4.9** **had a significantly lower survival time** with a **significant difference in median survival** between groups. **Log-rank test p-value**: 0.017 is **Statistically significant**, suggesting that higher core to knee temperature correlates with lower survival in septic shock patients.


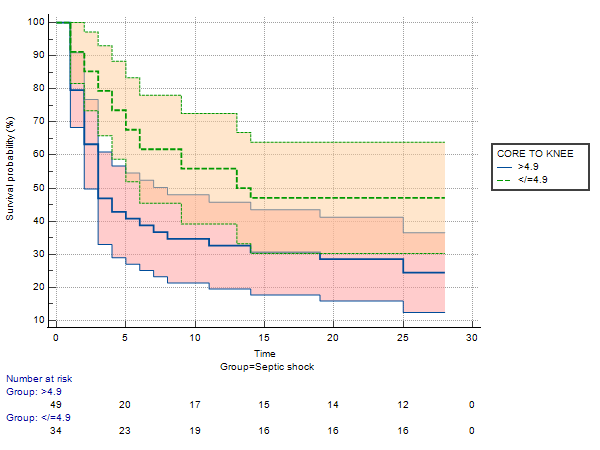


**Table S10:** Mean survival times in patients with septic shock taking a zero-hour cutoff of core to knee temperature gradient at 4.9°F.

| Core to Knee | Mean | SE | 95% CI for the mean | Median | 95% CI for the median |
| --- | --- | --- | --- | --- | --- |
| >4.9 | 10.490 | 1.615 | 7.324 to 13.656 | 3.000 | 2.000 to 7.000 |
| </=4.9 | 16.147 | 1.986 | 12.255 to 20.039 | 13.000 | 5.000 to 14.000 |
| Overall | 12.807 | 1.290 | 10.279 to 15.336 | 6.000 | 3.000 to 14.000 |

**GRAY ZONE ANALYSIS**

Since no threshold achieved a post-test probability of 95% for ruling in mortality, an alternative ap proach was used. The gray zone was selected to ensure that the percentage of subjects classified within the gray zone is minimized, thereby improving clinical applicability.

**Figure S10:** Gray zone analysis of Core to knee temperature gradient at zero hours with 48-hour, 7 day and 28 day mortality.

7-Day Mortality: Gray zone range: 4.57 to 12.4°F, with 65.34% of subjects classified as inconclusive.

28-Day Mortality: Gray zone range: 4.93 to 9.2°F, with 43.75% of subjects in the gray zone.

48-Hour Mortality: Gray zone range: 4.33 to 9.95°F, with 61.93% of subjects classified as uncertain

**
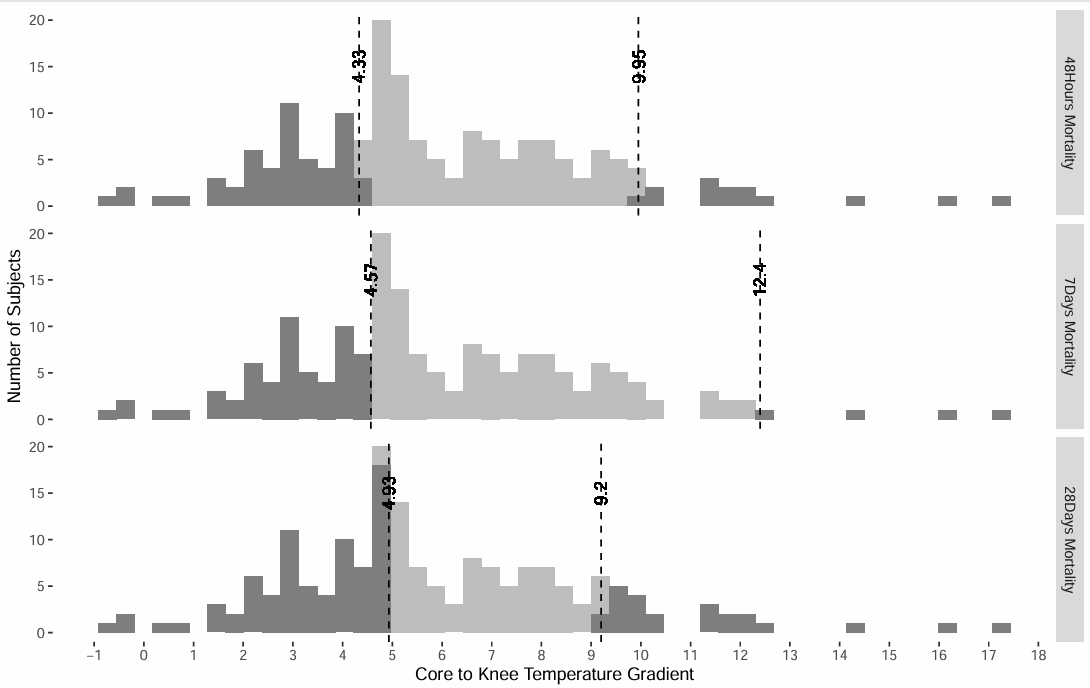
**

**Figure S 11:** Gray zone analysis of Core to toe temperature gradient at zero hours with 48-hour, 7 day and 28 day mortality.

7-Day Mortality: Gray zone range: 7.22 to 13.93°F, with 54.55% of subjects in the gray zone.

28-Day Mortality: Gray zone range: 3.05 to 16.4°F, with 84.09% of subjects in the gray zone.

48-Hour Mortality: Gray zone range: 3.05 to 19.7°F, with 95.45% of subjects in the gray zone**.**

**
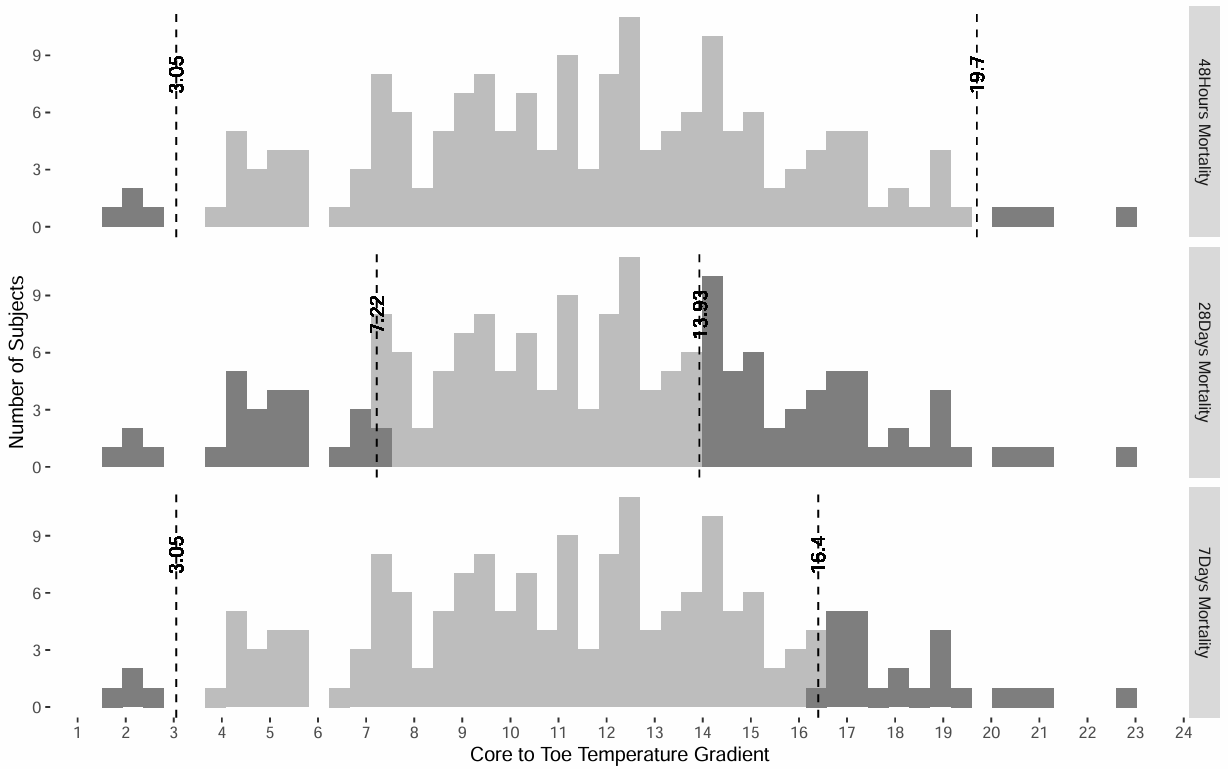
**
